# $\dot{V}$ O_2max_ and the kinetics of $\dot{V}$ O_2_, muscle oxygen delivery, and muscle deoxygenation

**DOI:** 10.3389/fphys.2025.1656980

**Published:** 2025-12-11

**Authors:** Gabriele Marinari, Darren S. DeLorey

**Affiliations:** Faculty of Kinesiology, Sport, and Recreation, University of Alberta, Edmonton, AB, Canada

**Keywords:** maximal oxygen uptake, aerobic fitness, oxygen uptake kinetics, blood flow kinetics, deoxyhemoglobin kinetics, oxidative phosphorylation, oxygen delivery

## Abstract

**Introduction:**

Aerobic fitness and oxygen uptake kinetics (τ $\dot{V}$ O_2_) at the onset of exercise appear to be inversely correlated, however, the mechanisms underlying changes in τ $\dot{V}$ O_2_ across different levels of aerobic fitness have not been elucidated. The purpose of this study was to investigate the relationship between maximal $\dot{V}$ O_2_ ( $\dot{V}$ O_2max_) and τ $\dot{V}$ O_2_ and determine whether the capacity to deliver or to utilize O_2_ limits τ $\dot{V}$ O_2_ in an aerobic fitness dependent manner.

**Methods:**

Twenty-three healthy, young males (25 ± 4 years) with a $\dot{V}$ O_2max_ classified as superior (S; $\dot{V}$ O_2max_ > 60 mL**·**kg^−1^ **·**min^−1^, n = 7), good (G; $\dot{V}$ O_2max_ = 45-55 mL**·**kg^−1^ **·**min^−1^, n = 8) or poor (P; $\dot{V}$ O_2max_ < 40 mL**·**kg^−1^ **·**min^−1^, n = 8) performed two moderate-intensity knee-extension (KE) exercise transitions (80% of gas exchange threshold) on a custom-built KE ergometer. $\dot{V}$ O_2_ was measured breath-by-breath. Leg blood flow (BF) was measured by doppler ultrasound at the femoral artery, and leg vascular conductance (LVC) was calculated as BF·mean arterial pressure (MAP)^−1^. Near-infrared spectroscopy derived-[HHb] was measured on the vastus lateralis muscle. τ $\dot{V}$ O_2_, τLVC, and τ[HHb] data were averaged and fit with a mono-exponential function.

**Results:**

τ $\dot{V}$ O_2_ was faster in the S (*P* < 0.01) and G (*P* < 0.05) fitness groups compared with the P fitness group. τ[HHb] was faster in the S (*P* < 0.05) compared with the P fitness group. $\dot{V}$ O_2max_ was inversely correlated to τ $\dot{V}$ O_2_ (*r* = −0.71; *P* < 0.001) and τ[HHb] (*r* = −0.55; *P* < 0.01), but not with τLVC (*r* = −0.12; *P* > 0.05). τ $\dot{V}$ O_2_ was positively correlated with τ[HHb] (*r* = -0.57; *P* < 0.01), but not with τLVC (*r* = −0.25; *P* > 0.05).

**Conclusion:**

$\dot{V}$ O_2max_ and τ $\dot{V}$ O_2_ were inversely correlated across fitness levels. These findings indicate that O_2_ delivery is not rate-limiting for τ $\dot{V}$ O_2_ across fitness levels and suggest that the intracellular capacity to utilize O_2_ may be the primary limiting factor for τ $\dot{V}$ O_2_ in healthy young adults, regardless of aerobic fitness.

## Introduction

In response to moderate-intensity step-transition exercise, oxygen uptake ( $\dot{V}$ O_2_) increases in a mono-exponential manner (Margaria et al., 1965; Margaria et al., 1963; Poole and Jones, 2012). The increase in $\dot{V}$ O_2_ is closely related to the metabolic demand of exercise and is achieved through an integrated response of the pulmonary, cardiovascular, and muscle metabolic systems (Grassi, 2003; Poole et al., 2007; Grassi et al., 2003; Grassi, 2005; Grassi et al., 2021; Rossiter, 2011). The rate at which $\dot{V}$ O_2_ adjusts at the onset of exercise, commonly referred to as $\dot{V}$ O_2_ kinetics (τ $\dot{V}$ O_2_), varies across individuals/populations and serves as an important index of aerobic metabolic function, with implications for metabolic stability (Grassi et al., 2015; Grassi et al., 2011; Grassi et al., 1996) and exercise tolerance (Poole and Jones, 2012; Grassi et al., 2011; Goulding et al., 2021).

Convective and diffusive O_2_ delivery to active muscles involves a complex interaction between motor unit recruitment, cardiac output, sympathetic vasoconstriction, and local vasodilation that matches muscle blood flow (BF) to metabolism (Pittman, 2016; Pittman, 2011; Poole et al., 2011; Poole and Musch, 2023; Poole et al., 2022; Marinari et al., 2025; DeLorey and Clifford, 2022; Joyner and Casey, 2015; Zoladz et al., 2016). Previous studies in healthy active individuals have reported that bulk leg BF increases at a rate similar to or faster than τ $\dot{V}$ O_2_ (Macdonald et al., 1998; Fukuba et al., 2004; Nyberg et al., 2017; duManoir et al., 2010; DeLorey et al., 2007; MacPhee et al., 2005; Koga et al., 2005; Endo et al., 2005; Harper et al., 2006; Jones et al., 2012; Schlup et al., 2015; Paterson et al., 2005; Love et al., 2023), suggesting that bulk O_2_ delivery may not be a limiting factor for τ $\dot{V}$ O_2_. In contrast, in disease states where pulmonary and cardiovascular function may be impaired and muscle O_2_ delivery is significantly slowed, τ $\dot{V}$ O_2_ is also slowed (Poole and Jones, 2012; Poole et al., 2007), suggesting that there may be a critical rate of O_2_ delivery required to support an increase in $\dot{V}$ O_2_ at the onset of exercise. However, measures of bulk leg BF do not provide any information related to the distribution of leg BF and may not reflect BF/O_2_ delivery to active muscle(s). Near-infrared spectroscopy (NIRS)-derived [deoxyhemoglobin] ([HHb]) provides an index of changes in muscle O_2_ extraction in response to exercise in a discrete region of the active muscle microcirculation (Grassi et al., 2003; DeLorey et al., 2003; Grassi and Quaresima, 2016). Consistent with the Fick equation, changes in [HHb] reflect the balance between changes in microvascular O_2_ delivery and those in muscle $\dot{V}$ O_2_. A faster τ[HHb] (i.e., O_2_ extraction) relative to τ $\dot{V}$ O_2_ may result from either slowed muscle O_2_ delivery relative to muscle $\dot{V}$ O_2_ or faster muscle $\dot{V}$ O_2_ relative to the rate of muscle O_2_ delivery. Previous studies have reported that [HHb] adapts at a faster rate than $\dot{V}$ O_2_ in healthy individuals (Grassi et al., 2003; DeLorey et al., 2003; Adami et al., 2011), demonstrating a mismatch between microvascular O_2_ delivery and muscle $\dot{V}$ O_2_ at the onset of exercise. The faster τ[HHb] relative to τ $\dot{V}$ O_2_ observed in these studies could reflect the following: (i) a slowed O_2_ delivery relative to the metabolic demand, with O_2_ delivery potentially limiting τ $\dot{V}$ O_2_; (ii) a faster O_2_ intracellular utilization relative to O_2_ delivery, with the slower O_2_ delivery not being rate-limiting for τ $\dot{V}$ O_2_. Unfortunately, these studies did not include measures of leg BF/O_2_ delivery to facilitate resolution of the underlying cause of the faster τ[HHb].

Individuals with high aerobic fitness appear to have faster τ $\dot{V}$ O_2_ than those with low aerobic fitness (Caputo et al., 2003; Caputo and Denadai, 2004; Cerretelli et al., 1979; George et al., 2018; Grey et al., 2015; Inglis et al., 2021; Koppo et al., 2004a), and an inverse relationship between maximal oxygen uptake ( $\dot{V}$ O_2max_) and τ $\dot{V}$ O_2_ has been reported (Inglis et al., 2021; Murias et al., 2011a; Fawkner et al., 2002; Chilibeck et al., 1996; Murgatroyd et al., 2011; Norris and Petersen, 1998; Powers et al., 1985). Although it seems unlikely that $\dot{V}$ O_2max_ and τ $\dot{V}$ O_2_ have a direct mechanistic basis, the relationship between $\dot{V}$ O_2max_ and τ $\dot{V}$ O_2_ suggests that aerobic fitness may influence the physiological determinants of τ $\dot{V}$ O_2_.

Therefore, the purpose of this study was to investigate the following hypotheses: 1) $\dot{V}$ O_2max_ and τ $\dot{V}$ O_2_ would be inversely correlated; and 2) the limiting factor to τ $\dot{V}$ O_2_ would be a function of $\dot{V}$ O_2max_, with O_2_ delivery limiting τ $\dot{V}$ O_2_ in participants with “poor” aerobic fitness, but not in participants with “good” and “superior” aerobic fitness.

## Methods

### Participants

This study was approved by the University of Alberta Health Sciences Research Ethics Board (Pro00015860). Twenty-three healthy young male individuals volunteered and provided written informed consent to participate in the study (Table 1). Participants were not undertaking any training program during the study period and were stratified into three aerobic fitness groups based on their relative $\dot{V}$ O_2max_, according to the American College of Sports Medicine for young male individuals (ages 20–29 years) (Kaminsky et al., 2015), as follows: “poor” (P; $\dot{V}$ O_2max_ < 40 mL·kg^−1^·min^−1^, n = 8), “good” (G; $\dot{V}$ O_2max_ = 45–55 mL·kg^−1^·min^−1^, n = 8), and “superior” (S; $\dot{V}$ O_2max_ > 60 mL·kg^−1^·min^−1^, n = 7). All subjects were non-obese (BMI <30 kg·m^−2^), non-smokers, and free from any previously diagnosed respiratory, cardiovascular, metabolic, or musculoskeletal disease. No subjects were using medications known to alter the cardiorespiratory response to exercise during this study.

**TABLE 1:** Participant characteristics.

	Superior (*n* = 7)	Good (*n* = 8)	Poor (*n* = 8)
Age (years)	25 ± 5	24 ± 4	26 ± 2
Height (cm)	181 ± 7	181 ± 6	180 ± 6
Body mass (kg)	76.1 ± 11.7	74.1 ± 7.5	79.7 ± 8.6
$\dot{V}$ O_2max_ (mL·kg^−1^·min^−1^)	68.5 ± 3.6*	50.5 ± 3.0^*^	32.8 ± 4.8^*^
$\dot{V}$ O_2max_ (L·min^−1^)	5.17 ± 0.54^*^	3.75 ± 0.49^*^	2.60 ± 0.43^*^
KE WR_peak_ (W)	84 ± 41	78 ± 19	56 ± 15
KE WR moderate (W)	47 ± 11^*^	33 ± 9^*^	23 ± 7^*^
KE $\dot{V}$ O_2peak_ (mL·kg^−1^·min^−1^)	32.3 ± 4.4^*^	27.6 ± 4.9^*^	20.8 ± 4.9^*^
KE $\dot{V}$ O_2peak_ (L·min^−1^)	2.41 ± 0.47^*^	2.05 ± 0.46^*^	1.63 ± 0.34^*^

Values are mean ± standard deviation. $\dot{V}$ O_2max_, maximal oxygen uptake; $\dot{V}$ O_2peak_, peak oxygen uptake; WR, work rate; W, watts; KE, knee extension.

^*^Indicates significant difference (*P* < 0.05) between all aerobic fitness groups.

## Experimental protocol

All testing was completed in the Integrative Human Exercise Physiology Laboratory at the University of Alberta. Participants reported to the laboratory on three separate occasions. Participants were instructed to abstain from exercise, caffeine, alcohol, and ibuprofen for 24 h prior to testing and to eat a light meal ∼2 h before exercise testing. Laboratory temperature was maintained between 20 °C and 22 °C.

*Day 1.* Participants completed an incremental exercise test to volitional exhaustion on a cycle ergometer (Ergoselect 200 K, Ergoline, Bitz, Germany) to determine $\dot{V}$ O_2max_. Following 2 min of baseline data collection, participants began pedaling, and the work rate was progressively increased in a ramp-like fashion at 30 watts (W)·min^−1^ to volitional exhaustion. Criteria used to establish a maximal test included the observation of a plateau in peak $\dot{V}$ O_2_, despite an increased work rate, a respiratory exchange ratio (RER) > 1.1, achievement of >90% age-predicted maximal heart rate (HR), and volitional exhaustion. Participants were then assigned to pre-determined aerobic fitness groups described above.

*Day 2.* Participants completed an incremental alternate-leg knee-extension (KE) exercise test to volitional exhaustion on a custom-built KE ergometer, as previously described (DeLorey et al., 2007). This test was conducted to determine individual work rates for moderate-intensity KE exercise. After 2 min of resting baseline data collection, participants completed 1-min of passive (unloaded) KE exercise, followed by alternate-leg KE exercise at a cadence of 30 contractions per leg per minute (cpm) from an initial work rate of 18 W. The work rate was then increased 3 W·min^−1^ until volitional exhaustion or until participants were unable to maintain a cadence of 30 cpm. Criteria used to establish a maximal test were a plateau in $\dot{V}$ O_2_ despite an increase in the work rate, an RER >1.10, and volitional exhaustion. The gas exchange threshold (GET) was defined as the $\dot{V}$ O_2_ at which CO_2_ production ( $\dot{V}$ CO_2_) began to increase disproportionately with respect to $\dot{V}$ O_2_, concurrent with an increase in the ratio of minute ventilation ( $\dot{V}$ E) to $\dot{V}$ O_2_ and end-tidal PO_2_, while $\dot{V}$ E/ $\dot{V}$ CO_2_ and end-tidal PCO_2_ remained stable. Moderate-intensity KE exercise was defined as a work rate corresponding to 80% of the work rate at the GET.

*Day 3.* Participants completed two step transitions from passive KE exercise to moderate-intensity KE exercise to determine the on-transient τ $\dot{V}$ O_2_, τ[HHb], and τLVC. Testing began with 2 min of resting baseline data collection, followed by 1-min of passive KE exercise. Thereafter, the work rate was increased in a stepwise manner, and participants performed 5 min of constant-load moderate-intensity KE exercise. Following a 15-min recovery period, the protocol was repeated, and the data from the two exercise transitions were ensemble-averaged (see *data analysis*). All KE exercise was performed at a cadence of 30 cpm and was preceded by 1-min of passive, unloaded exercise to minimize the effects of mechanical inertia and muscle mechanical factors and to increase the amplitude of the on-transient responses, thereby improving the signal-to-noise ratio and the accuracy of kinetics analysis. Participants’ legs were secured to the lever arms of the KE ergometer to facilitate passive exercise.

## Measurements

For all exercise testing, participants breathed through a mouthpiece, with their nose occluded. A low-resistance mass-flow meter was used to measure pulmonary gas exchange ( $\dot{V}$ O_2_, $\dot{V}$ CO_2_, and RER) and $\dot{V}$ E breath-by-breath via open-circuit indirect calorimetry (Vmax® 229d; Viasys™ Healthcare, Palm Springs, CA). Prior to each test, the flow meter was calibrated with a 3-L calibration syringe, and the O_2_ and CO_2_ analyzers were calibrated with gases of known concentrations.

A three-lead electrocardiogram (ECG) was measured continuously (Power Laboratory 16/30, AD Instruments, Colorado Springs, CO), and HR was derived from the ECG. Beat-by-beat arterial blood pressure (BP) was measured using photoplethysmography on the middle finger of the right hand (Finometer™, Finapres Medical Systems, Amsterdam, Netherlands). BP was also measured using a sphygmomanometer, and Finometer BP was corrected to manually measured pressures when pressure differences were observed. Mean arterial pressure (MAP) was calculated on a beat-by-beat basis.

Mean blood velocity (MBV) of the right femoral artery was measured using pulsed-Doppler ultrasonography (Vivid I, General Electric, Waukesha, WI). Data were acquired continuously using a 7.5 MHz probe positioned 2–3 cm distal to the inguinal ligament and proximal to the femoral artery bifurcation, while the probe was maintained at a 45-degree angle of insonation. Prior to exercise testing on days 2 and 3, the resting diameter of the femoral artery was measured in triplicate during diastole. The three measures were then averaged to determine the baseline femoral artery diameter. Previous studies have demonstrated that the common femoral artery diameter does not change from resting values during exercise. Thus the resting diameter was used for blood flow calculations during exercise (Macdonald et al., 1998; MacPhee et al., 2005; Paterson et al., 2005; Rådegran and Saltin, 2000). Mean blood velocity in cm·s^−1^ was measured on a beat-by-beat basis. Limb BF was calculated as BF (mL·min^−1^) = MBV**·**π**·** *r* ^2^·60, where *r* is the measured radius of the femoral artery. LVC was then calculated as follows: LVC (L·min^−1^·mmHg^−1^) = BF·MAP^−1^. Data were recorded using a PowerLab 16/30 system and Chart 7 data acquisition software (AD Instruments) at a sampling frequency of 100 Hz.

Relative [HHb] was measured in the vastus lateralis (VL) muscle using NIRS (NIRO 300, Hamamatsu Photonics, Hamamatsu, Japan), as described previously (DeLorey et al., 2007). In short, optodes were placed on the belly of the VL at the midpoint between the lateral epicondyle and the greater trochanter of the femur. These optodes were contained within an optically dense plastic holder to minimize extraneous light and the loss of NIR light from the field of interrogation and ensure the position of optodes relative to each other. This optode assembly was affixed to the skin using tape and was wrapped in an elastic bandage to further prevent movement of the optodes and interference of extraneous light.

The intensities of incident and transmitted light were continuously recorded, along with relevant extinction coefficients and estimated optical path length, assuming a differential path length factor of 3.83 (DeLorey et al., 2007). These values were used for online estimation and display of changes in concentrations of oxyhemoglobin (O_2_Hb), HHb, and total hemoglobin (Hb_tot_). The raw attenuation signal in optical density units was sampled at 1Hz and transferred to a computer and stored for future analysis. Prior to testing, the NIRS unit was “zeroed” to a stable, resting baseline.

### Data analysis

#### τ $\dot{V}$ O_2_

Breath-by-breath $\dot{V}$ O_2_ data were filtered for aberrant data points, interpolated to 1-s intervals and then ensemble-averaged into 5-s time bins to yield a single response for each subject. The on-transient responses were modeled using nonlinear, least squares regression procedures (OriginLab, Northampton, MA, United States), with a mono-exponential function:

$$Y_{\left(t\right)}=Y_{\left(b\right)}+A\;\left\{1-e^{\left[-\left(t-\text{TD}\right)/\tau \right]}\right\},$$ (1)

where *Y* represents $\dot{V}$ O_2_ at any time (*t*), *b* is the baseline value of Y at the point in time from which the data were fit, A is the amplitude of the increase in *Y* above the baseline value, τ is the time constant defined as the duration of time at which *Y* increases to a value equivalent to 63% of A, and TD is the time delay. Only the primary component (*phase II*) of the on-transient $\dot{V}$ O_2_ response was included in the fitting window, excluding the so-called cardio dynamic component (*phase I*). *Phase I* was identified by extending the fitting window backward from ∼40 s until τ, χ^2^, and confidence interval (CI) began to increase, as described elsewhere (Love et al., 2023; Rossiter et al., 2001).

##### τLVC

Similar to $\dot{V}$ O_2_, LVC data were filtered for aberrant data points, interpolated to 1-s intervals and then ensemble-averaged into 5-s time bins to yield a single response for each subject. Subsequently, LVC data were fit with a mono-exponential model as described in Equation 1 from the onset of exercise to either the end of the exercise or a potential peak (overshoot) manifested within the first minutes of exercise (Love et al., 2023).

##### τ[HHb]

Similarly, [HHb] data were ensemble-averaged into 5-s time bins to yield a single response for each subject. Subsequently, [HHb] data were fit with a mono-exponential model as described in Equation 1 from the end of the calculated TD (CTD), representing the first value following the exercise onset at which [HHb] began to systematically increase. Thereafter, [HHb] was fit (i) to the end of exercise in the case of stable responses, (ii) to a potential overshoot within the first seconds/minutes of exercise, or (iii) to the point preceding a potential gradual increase in the response following an initial steady-state behavior (Love et al., 2023).

Following the observation of a consistent “overshoot” in [HHb] at the onset of exercise in the S fitness group, further analyses were performed to quantify its magnitude and make inferences about microvascular and intracellular oxidative responses across groups. A 30-s moving average was performed within the first 3 minutes of exercise of the normalized [HHb] for all groups. Thereafter, the time points at which the highest 30-s moving average occurred served to identify the time window with higher [HHb] overshoot incidence, which ranged from 40 s to 120 s of the exercise on-transient. The difference between the average [HHb] between 40 s and 120 s and the last minute of [HHb] during exercise was computed for each subject and compared across groups.

##### Relationships between kinetics and $\dot{V}$ O_2max_

Correlation analyses were performed to examine the relationship between τ $\dot{V}$ O_2_, τLVC, and τ[HHb] with varying $\dot{V}$ O_2max_ and elucidate the relationship between O_2_ delivery (i.e., τLVC) and extraction (i.e., τ[HHb]) with varying τ $\dot{V}$ O_2_.

##### Gain

The gain for $\dot{V}$ O_2_, LVC, and [HHb] was computed as the amplitude of the response of each variable divided by the work rate amplitude of each subject ( $\dot{V}$ O_2GAIN_, mL·min^−1^·W^−1^; LVC_GAIN_, mL·min^−1^·mmHg^−1^·W^−1^; [HHb]_GAIN_, µM·min^−1^·W^−1^).

### Statistical analysis

All data are reported as the mean ± standard deviation. A one-way ANOVA was performed for between-group comparisons of $\dot{V}$ O_2max_, gain, τ $\dot{V}$ O_2_, τLVC, and τ[HHb]. Within group differences between τ $\dot{V}$ O_2_, τLVC and τ[HHb] were determined by one-way ANOVA. When significant F-ratios were found, Tukey’s HDS *post hoc* analysis was performed. A paired Student’s t-test was performed to compare a potential initial [HHb] overshoot within groups. Relationships between variables were determined using Pearson’s *r* correlation. A *p*-value <0.05 was considered statistically significant. Statistical analyses were performed using SPSS (v. 29.0, IBM, Chicago, United States).

## Results

### Participant characteristics

Participant characteristics are reported in Table 1. Consistent with the design of the study, both absolute and relative $\dot{V}$ O_2max_ values were different between S, G and P fitness groups (*p* < 0.001). Similarly, absolute and relative KE $\dot{V}$ O_2peak_ were also different between the S, G and P fitness groups (*p* < 0.01). Additionally, peak and moderate KE work rates were significantly different across groups (all *p* < 0.01).

**FIGURE 1:**
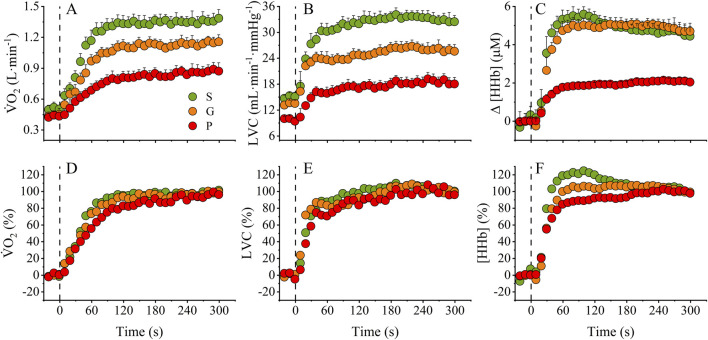
Absolute and normalized $\dot{V}$ O_2_, LVC, and [HHb] kinetics profiles. The upper panel shows the absolute profiles of $\dot{V}$ O_2_ **(A)**, LVC **(B)**, and [HHb] **(C)**, while the lower panel shows the normalized profiles as a function of baseline (0%) and end-exercise (100%) **(D–F)**. S, “superior” fitness; G, “good” fitness; P, “poor” fitness; $\dot{V}$ O_2_, oxygen uptake; LVC, leg vascular conductance; [HHb] de-oxyhemoglobin concentration; Δ, delta change.

Pre-transition baseline values for $\dot{V}$ O_2_, LVC, and [HHb] are reported in Table 2. Averaged absolute and normalized profiles for $\dot{V}$ O_2_, LVC, and [HHb] are depicted in Figure 1 (upper and lower panel, respectively).

**TABLE 2:** Baseline values.

Group	$\dot{V}$ O_2_ (L·min^−1^)	LVC (mL·min^−1^·mmHg^−1^)	[HHb] (µM)
S (*n* = 7)	0.49 ± 0.09	14.0 ± 0.5	-2.4 ± 0.8
G (*n* = 8)	0.44 ± 0.07	14.0 ± 0.4	-1.4 ± 1.4
P (*n* = 8)	0.46 ± 0.09	10.0 ± 0.3	-0.8 ± 1.2

Values are mean ± standard deviation. $\dot{V}$ O_2_, oxygen uptake; LVC, leg vascular conductance; [HHb], deoxyhemoglobin concentration; S, superior; G, good; P, poor.

#### τ $\dot{V}$ O_2_

**FIGURE 2:**
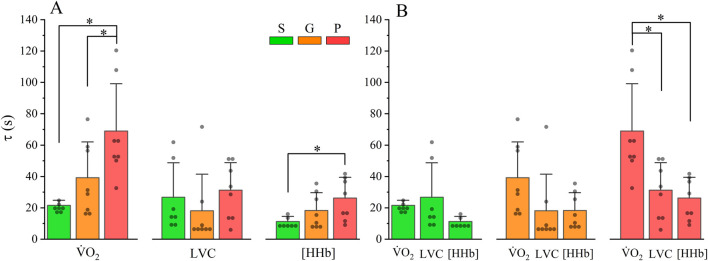
Between- **(A)** and within **(B)**-group comparisons of τ $\dot{V}$ O_2_, τLVC, and τ[HHb]. Black lines above the bar graphs indicate significant differences (*p* < 0.05). S, “superior” fitness; G, “good” fitness; P, “poor” fitness. τ, time constant. Filled circles indicate individual data points.

τ $\dot{V}$ O_2_ values for S, G, and P were 22 ± 3 s (CI_95_: 8 s range), 39 ± 23 s (CI_95_: 11 s range), and 69 ± 30 s (CI_95_: 23 s range), respectively, and were different across the fitness groups (*p* < 0.05; Figure 2A). In particular, post hoc analysis revealed that τ $\dot{V}$ O_2_ in S and G was faster than τ $\dot{V}$ O_2_ in P (*p* < 0.01 and *p* < 0.05, respectively), whereas τ $\dot{V}$ O_2_ was not different between S and G (*p* > 0.05).

#### τLVC

τLVCs for S, G, and P were 27 ± 22 s (CI_95_: 9 s range), 18 ± 23 s (CI_95_: 7 s range), and 31 ± 18 s (CI_95_: 16 s range), respectively, and were not significantly different (*p* > 0.05; Figure 2A).

#### τ[HHb]

The CTDs for S, G, and P, from which the exponential fit began, were 13 ± 4 s, 12 ± 6 s, and 12 ± 5 s, respectively, and not significantly different (*P* > 0.05). τ[HHb] values for S, G, and P were 11 ± 3 s (CI_95_: 3 s range), 18 ± 11 s (CI_95_: 5 s range), and 26 ± 13 s (CI_95_: 4 s range), respectively, and were different between fitness groups (*P* < 0.05; Figure 2A). In particular, post hoc analysis revealed that τ[HHb] was faster in S compared with P (*p* < 0.05), whereas no significant difference was observed in τ[HHb] between S and G (*p* > 0.05: Figure 2A).

An overshoot in [HHb] in the S fitness group was confirmed (*p* < 0.05), whereas no [HHb] overshoot was observed for the G or P fitness group.

#### Within-group kinetics

Comparison between τ $\dot{V}$ O_2_, τLVC, and τ[HHb] is depicted in Figure 2B. No within-group differences between τ $\dot{V}$ O_2_, τLVC, and τ[HHb] were observed for S and G fitness groups (*p* > 0.05), whereas a within-group difference in the P fitness group was detected (*p* < 0.05). In particular, post hoc analysis revealed that τLVC and τ[HHb] were faster (p < 0.01) than τ $\dot{V}$ O_2_ in P (*p* < 0.05).

#### Correlations

**FIGURE 3:**
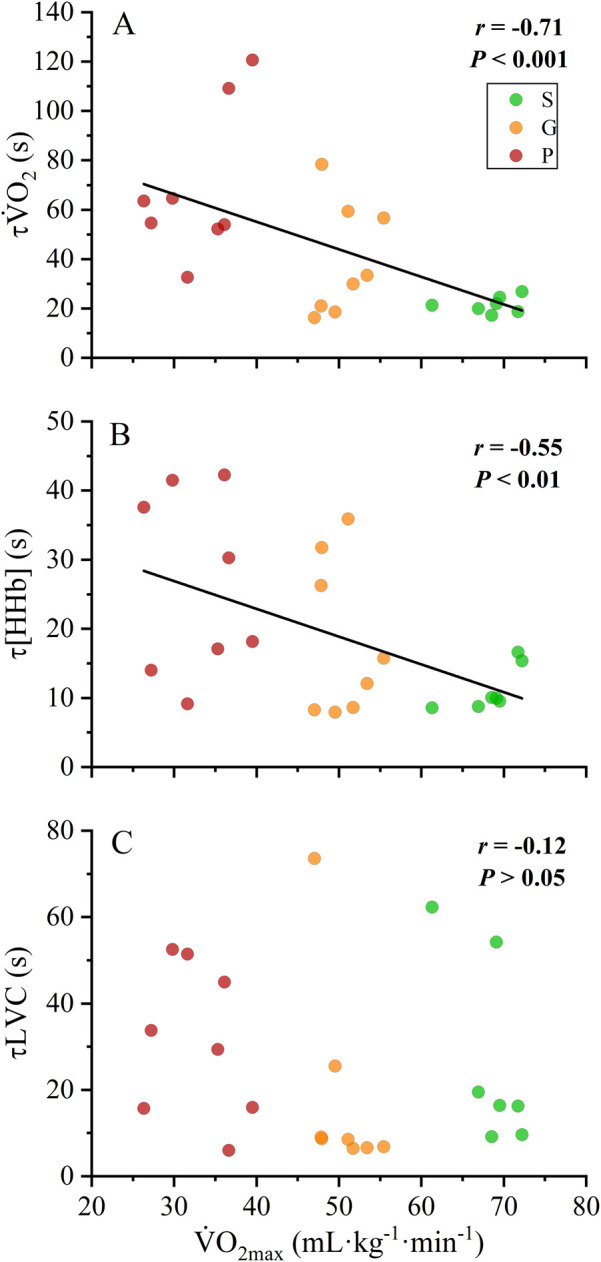
Between-group correlations of $\dot{V}$ O_2max_ with τ $\dot{V}$ O_2_ **(A)**, τ[HHb] **(B)** and τLVC **(C)**. S, “superior” fitness; G, “good” fitness; P, “poor” fitness; $\dot{V}$ O_2max_, maximal oxygen uptake; τ $\dot{V}$ O_2_, oxygen uptake kinetics; τLVC, leg vascular conductance kinetics; τ[HHb] de-oxyhemoglobin concentration kinetics. Black lines indicate significant correlations (*p* < 0.05) across groups.

Correlations of $\dot{V}$ O_2max_ with τ $\dot{V}$ O_2_, τLVC, and τ[HHb] are displayed in Figure 3 (A, B, and C). When the data from all groups were pooled, both τ $\dot{V}$ O_2_ (*r* = −0.71; *p* < 0.001) and τ[HHb] (*r* = −0.57; *p* < 0.01) showed a significant negative correlation with $\dot{V}$ O_2max_, whereas τLVC and $\dot{V}$ O_2max_ were not correlated (*r* = −0.12; *p* > 0.05). All within-group correlations were not significant (all *p* > 0.05).

**FIGURE 4:**
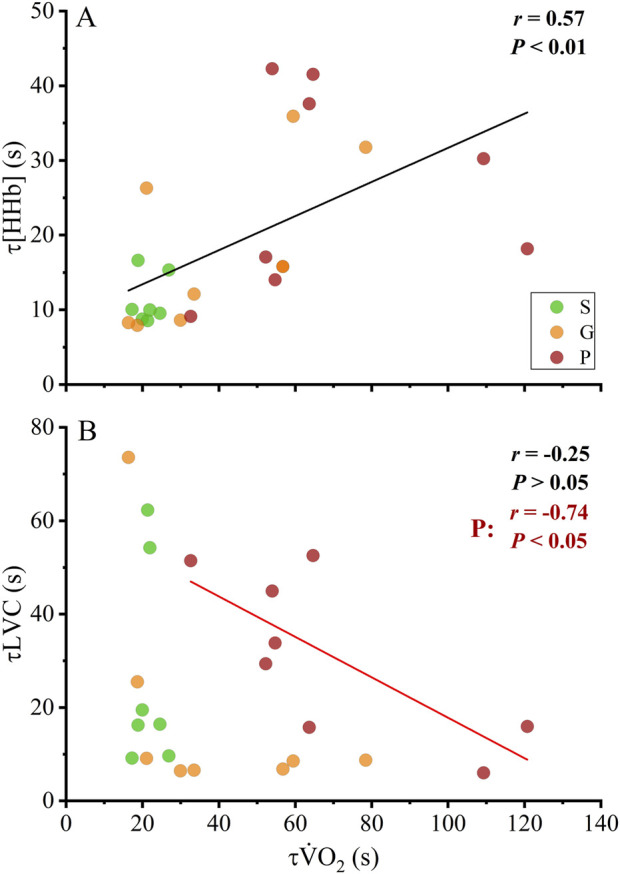
Between- and within-group correlations of τ $\dot{V}$ O_2_ with τ[HHb] **(A)** and τLVC **(B)**. S, “superior” fitness; G, “good” fitness; P, “poor” fitness; τ $\dot{V}$ O_2_, oxygen uptake kinetics; τLVC, leg vascular conductance kinetics; τ[HHb] de-oxyhemoglobin concentration kinetics. Black lines indicate significant correlations (*p* < 0.05) across groups. The red line indicates a significant correlation within the P fitness group.

Correlations of τ $\dot{V}$ O_2_ with τLVC and τ[HHb] are depicted in Figures 4A,B. When the data from all groups were pooled, a significant positive correlation was found between τ[HHb] and τ $\dot{V}$ O_2_ (*r* = 0.57; *p* < 0.01), whereas τLVC and τ $\dot{V}$ O_2_ were not correlated (*r* = −0.25; *p* > 0.05). Within-group correlations were all non-significant (all *p* > 0.05), except for a negative correlation between τ $\dot{V}$ O_2_ and τLVC in the P fitness group (*r* = −0.74; *p* < 0.05).

#### Gains

The gain for all variables are reported in Table 3. No differences in $\dot{V}$ O_2GAIN_ (*p* > 0.05), LVC_GAIN_ (*p* > 0.05), and [HHb]_GAIN_ (*p* > 0.05) were observed between fitness groups.

**TABLE 3:** Gain values.

Group	$\dot{V}$ O_2GAIN_ (mL·min^−1^·W^−1^)	LVC_GAIN_ (mL·min^−1^·mmHg^−1^·W^−1^)	[HHb]_GAIN_ (µM·min^−1^·W^−1^)
S (*n* = 7)	18.8 ± 2.2	0.39 ± 0.19	0.10 ± 0.06
G (*n* = 8)	21.5 ± 4.3	0.39 ± 0.23	0.16 ± 0.10
P (*n* = 8)	20.7 ± 3.5	0.48 ± 0.56	0.09 ± 0.05

Values are mean ± standard deviation. $\dot{V}$ O_2GAIN_, oxygen uptake gain; LVC_GAIN_, leg vascular conductance gain; [HHb]_GAIN_, deoxyhemoglobin concentration gain. S, superior; G, good; P, poor.

## Discussion

The purpose of the present study was to investigate the relationship between τ $\dot{V}$ O_2_ and $\dot{V}$ O_2max_, and determine whether the capacity to deliver or to utilize O_2_ limits τ $\dot{V}$ O_2_ in an aerobic fitness-dependent manner. $\dot{V}$ O_2max_ was inversely correlated with τ $\dot{V}$ O_2_, and τ $\dot{V}$ O_2_ was faster in the S and G fitness groups than in the P fitness group. τLVC was not different between groups, and it was not positively correlated with $\dot{V}$ O_2max_ or τ $\dot{V}$ O_2_ across and within groups, indicating that O_2_ delivery kinetics were similar between groups despite large differences in aerobic fitness. τ[HHb] was faster in the S than in the P fitness group and was inversely correlated with $\dot{V}$ O_2max_ and positively correlated with τ $\dot{V}$ O_2_ across groups. Collectively, these data indicate that O_2_ delivery was not limiting for τ $\dot{V}$ O_2_ and that intracellular oxidative metabolism may limit τ $\dot{V}$ O_2_ in healthy individuals, regardless of aerobic fitness.

In the present study, τ $\dot{V}$ O_2_ was slower in the P than in the G and S fitness groups, whereas τ $\dot{V}$ O_2_ was not different between the S and G fitness groups. Consistent with the present data, several cross-sectional studies have also reported faster τ $\dot{V}$ O_2_ in trained or active individuals than in untrained or sedentary individuals (Caputo et al., 2003; Caputo and Denadai, 2004; Cerretelli et al., 1979; George et al., 2018; Grey et al., 2015; Inglis et al., 2021; Koppo et al., 2004a), and although the data are not conclusive, an inverse relationship between $\dot{V}$ O_2max_ and τ $\dot{V}$ O_2_ has often been reported (Inglis et al., 2021; Murias et al., 2011a; Fawkner et al., 2002; Chilibeck et al., 1996; Murgatroyd et al., 2011; Norris and Petersen, 1998; Powers et al., 1985). Inglis et al. (2021) investigated the relationship between $\dot{V}$ O_2max_ and τ $\dot{V}$ O_2_ in untrained ( $\dot{V}$ O_2max_ ∼ 40 mL·kg^−1^·min^−1^) and trained ( $\dot{V}$ O_2max_ ∼ 58 mL·kg^−1^·min^−1^) participants and reported an inverse relationship between $\dot{V}$ O_2max_ and τ $\dot{V}$ O_2_ within the untrained group, but not within the trained group. The kinetics of cardiac output (Q; measured through impedance cardiography) were not different between untrained and trained groups and were similar to or even faster than τ $\dot{V}$ O_2_ in both groups, suggesting that O_2_ delivery was not limiting in either group. Although Q kinetics do not provide information related to the distribution of Q or muscle blood flow, Inglis et al. (2021) reported a higher [HHb]/ $\dot{V}$ O_2_ ratio in the vastus lateralis muscle of the untrained group than that of the trained group, suggesting that microvascular O_2_ delivery may be slower within active muscle and contribute to a slower $\dot{V}$ O_2_ response in untrained participants. Whether τ $\dot{V}$ O_2_ is limited by O_2_ delivery (DeLorey et al., 2003; Hughson et al., 1996; Murias et al., 2011b; Murias et al., 2011c), intracellular oxidative metabolism (Zoladz et al., 2016; Grassi, 2000; Christensen et al., 2011; Korzeniewski and Rossiter, 2015), or a combination of both (Murias et al., 2014) within the active limbs across fitness levels remains controversial.

This study, in line with others (Macdonald et al., 1998; Fukuba et al., 2004; Nyberg et al., 2017; duManoir et al., 2010; DeLorey et al., 2007; MacPhee et al., 2005; Koga et al., 2005; Endo et al., 2005; Harper et al., 2006; Jones et al., 2012; Schlup et al., 2015; Paterson et al., 2005; Love et al., 2023; Inglis et al., 2021), indicated that O_2_ delivery (i.e., τLVC) was faster or as fast as τ $\dot{V}$ O_2_ within each fitness group. Furthermore, τLVC and LVC_GAIN_ were not different between groups despite large differences in aerobic fitness, and τLVC was not correlated with either τ $\dot{V}$ O_2_ or $\dot{V}$ O_2max_, suggesting that O_2_ delivery is not a rate-limiting factor for τ $\dot{V}$ O_2_ across fitness levels. Consistent with this notion, pump perfusion of canine muscle to eliminate temporal delays in O_2_ delivery and enhancement of muscle O_2_ diffusive capacity did not accelerate τ $\dot{V}$ O_2_ during moderate-intensity exercise (Grassi et al., 1998a; Grassi et al., 1998b).

Although the present study suggests that at the onset of exercise, O_2_ delivery to the active muscles is not limiting for τ $\dot{V}$ O_2_ across fitness levels (evidenced by similar τLVC and LVC_GAIN_ in all groups), it is important to acknowledge that LVC reflects bulk O_2_ delivery to the whole limb and does not reflect microvascular O_2_ delivery to active muscle fibers. Therefore, it could be argued that either better microvascular O_2_ distribution or intracellular oxidative mechanisms underlie differences in τ $\dot{V}$ O_2_ between fitness levels. If microvascular O_2_ delivery limits τ $\dot{V}$ O_2_ across fitness levels, an inverse relationship between τ[HHb] and τ $\dot{V}$ O_2_ should be observed, and faster τ[HHb] would be expected in the P fitness group (i.e., the group with the slowest τ $\dot{V}$ O_2_) than in the G and S fitness groups. However, τ[HHb] was significantly faster in the S fitness group than in the P fitness group and positively correlated with τ $\dot{V}$ O_2_ and negatively correlated with $\dot{V}$ O_2max_ across fitness groups. Potential contributions of microvascular O_2_ delivery to τ $\dot{V}$ O_2_ in our P fitness group, where τ[HHb] was faster than τ $\dot{V}$ O_2_, cannot be dismissed. The direction of the relationships between τ[HHb] with τ $\dot{V}$ O_2_ and $\dot{V}$ O_2max_, as well as the faster τ[HHb] in the S than in the P fitness group, and, more importantly, the similar τLVC and LVC_GAIN_ between groups support the notion that intracellular oxidative metabolism is the rate-limiting factor for τ $\dot{V}$ O_2_ across fitness levels.

Supporting enhanced intracellular oxidative metabolism in the S fitness group is the observed initial [HHb] overshoot. With τLVC not being limiting for τ $\dot{V}$ O_2_, the [HHb] overshoot may be attributed to a transient enhanced oxidative metabolism response and/or to specific motor unit recruitment strategies in highly trained individuals at the onset of exercise (Grassi et al., 2011; Marinari et al., 2025; Korzeniewski and Zoladz, 2003; Bowen et al., 2013; do Nascimento Salvador et al., 2023). Accordingly, it has recently been demonstrated that muscle excitation increases “disproportionally” at the onset of a step-transition exercise without prior warm-up in recreationally active individuals, which was connected to a greater [HHb] response (Marinari et al., 2025). A similar interpretation was proposed to explain the overshoot in $\dot{V}$ O_2_ observed in trained cyclists at intensities below the GET (Kilding and Jones, 2008; Koppo et al., 2004b).

Interestingly, end-exercise gains in $\dot{V}$ O_2_, LVC, and [HHb] were not different between fitness groups (Table 3), indicating similar adaptations at different levels of the O_2_ cascade system per unit work rate.

### Experimental considerations

In this study, $\dot{V}$ O_2_, LVC, and [HHb] were averaged over two trials. Increasing the number of trials would have likely improved the accuracy of our kinetics analyses. Nevertheless, the CI ranges of the parameter estimate “τ” in the S and G fitness groups were similar to those of a recent study where similar variables were averaged using five trials (Love et al., 2023). Our P fitness group reported greater variability for the estimation of τ $\dot{V}$ O_2_ and τLVC (but not for τ[HHb]), which may be caused by the reduced amplitude changes in $\dot{V}$ O_2_ and LVC due to smaller work rates. However, different $\dot{V}$ O_2_ and similar O_2_ delivery kinetics between fitness levels are in line with previous findings (Inglis et al., 2021), suggesting that our kinetics analyses were not significantly affected by the number of trials.

## Conclusion

In this study, $\dot{V}$ O_2max_ and τ $\dot{V}$ O_2_ were inversely correlated across three fitness levels. Although τ $\dot{V}$ O_2_ was faster in the “good” and “superior” fitness groups than in the “poor” fitness group, τLVC was similar between fitness groups and not correlated to either τ $\dot{V}$ O_2_ or $\dot{V}$ O_2max_. Conversely, τ[HHb] was inversely correlated to $\dot{V}$ O_2max_ and positively correlated to τ $\dot{V}$ O_2_ across fitness groups. Furthermore, τ[HHb] was faster in the “superior” fitness group than in the “poor” fitness group. Collectively, the present study suggests that O_2_ delivery is not a rate-limiting factor for τ $\dot{V}$ O_2_ and that the intracellular capacity to utilize O_2_ may be the primary limiting factor for τ $\dot{V}$ O_2_ in healthy individuals, regardless of aerobic fitness.

## Data Availability

The original contributions presented in the study are included in the article/supplementary material; further inquiries can be directed to the corresponding author.
